# Structure‐based vaccines targeting discontinuous A*β* epitopes prevent amyloid plaque deposition in male 5XFAD mice

**DOI:** 10.1002/alz70861_108030

**Published:** 2025-12-23

**Authors:** Aishwarya Sriraman

**Affiliations:** ^1^ Centre for prions and protein folding diseases, University of Alberta, Edmonton, AB Canada

## Abstract

**Background:**

Alzheimer's disease (AD) is thought to be caused by the misfolding of the amyloid beta (Aβ) and tau proteins, which both adopt beta‐sheet rich conformations and form oligomers and amyloid fibrils. Many attempts have been made to develop vaccines as prophylactics for AD employing these proteins in their linear form, but lacked structural specificity. Here, we present a novel approach to design vaccines based on the structures of Aβ fibrils that present their epitopes in a structurally‐controlled manner.

**Method:**

The innocuous HET‐s protein adopts a beta‐sheet rich conformation in its native state and was engineered to carry select Aβ surface epitopes in a discontinuous and structurally‐controlled manner. Vaccines were expressed in *E. coli*, purified, refolded, controlled for their structural fidelity, and used to immunize 5xFAD transgenic mice. The brains of unimmunized and immunized 5xFAD mice were collected at ∼200 days of age and analyzed for their Aβ plaque load.

**Result:**

As expected, unimmunized 5xFAD mice showed widespread Aβ plaque deposition throughout their cortex and hippocampus. 5xFAD mice immunized with unmodified HET‐s displayed no benefit and had essentially indistinguishable Aβ plaque loads from unimmunized animals. Mice that were immunized with the structure‐based vaccines had significantly reduced Aβ plaque deposits, but showed pronounced sex differences: female 5xFAD mice had a ∼50% overall reduction in their plaque load, while many male 5xFAD mice were essentially devoid of Aβ plaques.

**Conclusion:**

Structure‐based vaccines targeting surface‐exposed, discontinuous Aβ epitopes can prevent Aβ plaque deposition in male 5xFAD mice. However, immunized female 5xFAD mice display less benefit from the vaccines. Further investigations are needed to elucidate the underlying mechanisms for the observed sex differences and to increase the prophylactic efficacy of the novel vaccines.

